# Communications

**Published:** 1910-05

**Authors:** 


### COMMUNICATIONS

*To the Editor of the Journal-Record of Medicine,*
*1 to 5 South Broad Street, Atlanta, Ga.*

DEAR DOCTOR: Will you kindly publish the accompanying letter in the correspondence column of the next issue of the JOURNAL-RECORD OF MEDICINE?

This letter, already published with comments by the Editor in the Journal of the American Medical Association of March 12th, explains its object.

The necessity of publishing this letter under a key is obvious, as I would not care to have my name publicly connected with this unusual appeal. I would say, however, that all replies to this letter must go through my hands, as I will thus be able to save the family from the hands of quacks, imposters, etc.

Copy of your publication containing this letter will be greatly appreciated. Yours with thanks,

(Signed) A. A. ROSENBLOOM.

P. S.—Will gladly defray any expense incurred through handling any replies by means of the key, "Enquirer."

TO THE MEDICAL PROFESSION:

A gentleman of means, has a member of his family afflicted with Progressive Muscular Atrophy, the diagnosis having been with certainty established after consultation with some of the highest Neurological authorities of New York City and various cities of Europe.

These physicians are unanimously of the opinion that the case is incurable, inasmuch as up to the present, there has been published no form of treatment or medication which is known to have positively cured or arrested the inroads of this malady.

This gentleman wishes to spare no effort to bring relief. He believes that perhaps somewhere, some physician may have successfully hit upon some method of curing a case of Progressive Muscular Atrophy, but who through his inability to corroborate his results, owing to rarity of cases or through modesty, or for fear of being discredited, has failed to publish his case. This gentleman's idea, is to try and bring this record to the surface by making an appeal to the profession through this journal.

The case itself presents the characteristic picture and is typical of Progressive Muscular Atrophy in every particular. The patient is fifty years old; married; in excellent general health. About one and one-half years ago, the disease made its appearance in the left hand, progressed, and within a few months, involved the right hand. Its progress since has been very slow. The family of this patient wishes to announce that any physician who supplies a complete history and detailed description of the method of treatment of any case of Progressive Muscular Atrophy he may have successfully treated, the trial of which leads to the cure or arrest of the disease in their relative, will be rewarded by a liberal cash prize. Requests for further particulars and replies should be addressed to "Enquirer," care of this journal.

---

### REGULAR MEETING OF THE FULTON COUNTY MEDICAL SOCIETY.

April 7, 1910.

REPORTED BY R. R. DALY, M. D.

Dr. W. A. Crowe read a paper on "Some Abuses in Obstetrical Practice."

Dr. Earnest said that this subject should have more attention than it receives. Duties devolve upon us from the start of gestation; perfect measurements should surely be made in primiraræ to determine the possibility of their going to term. Next, general instructions to live in the open just as she has been in the habit of doing and not to stint herself in eating in order to keep the child small. The kidneys should be watched. Most of us do this in a perfunctory sort of way in the matter of albumen alone. The amount of urea should be known and the approximate amount of daily secretion should be observed. If it falls under the normal it should be increased to prevent eclampsia. Eclampsia *does* occur when there are no albumens and casts. Morphine should be used only when there is a great deal of pain and no dilatation of the cervix. He then gives enough to cause a night's rest, after which the cervix is likely to be soft. Used in this way there has been do damage to the offspring.

When should forceps be used? We are disposed to be in a hurry. We should give ample time, except in eclampsia. When the uterus is exhausted and relaxed, we should not wait just simply on a matter of time. The general practitioner should be acquainted with the best technique of the use of the forceps. The position of the head should be surely known and the axes of the pelvis well in mind. Chloroform should always be given with instrumental delivery, but too much has often caused the death of children. Ergot should never be given before delivery.

Dr. Olmsted said that morphine should be used in unyielding, thickened os with insufficient strength, especially in primiparæ. Sometimes just a few whiffs of chloroform gives the rest and lets the pains have a chance. At the eve of delivery he gives enough for anaesthesia. Used in this way he has never had any accident from the chloroform. He approves of caution in the use of forceps; there is too much haste in these days in the matter of the convenience of the doctor and it is inexcusable. If the woman has been in labor even two days and her condition is good, the forceps should not be used.

Dr. Frances Bradley said that the position of the woman during the puerperium was of the greatest importance; she should not be allowed to lie on her back, because of the danger of retroversions. Certain moderate muscular exercises during the puerperium were of value, not only in restoring the physiologic state, but in relieving the restlessness. The question of unskilled nurses and old granny remedies and relatives is hard for her to answer and these conditions stand much in her way even in these modern days.

Dr. Lokey said that the matter of vaginal secretions and the danger of ophthalmia neonatorum should be always kept in mind. This is most important to the child because 33 per cent. of the blindness in the country is due to carelessness in this regard.

Dr. Kime said the investigation of the kidneys is too frequently overlooked; the total amount of solids over all should be known. Toxic conditions from the alimentary canal impairs the kidneys so that they do not return to normal afterwards. Albumen is by no means enough to look for; the dieting should be kept from burdening the kidneys; meat and albumens should be kept down. Perineal repair should be something more than cosmetic. Buried, interrupted, layer cat-gut stitches should be used whenever there is a deep tear. As to the position in bed, he considered it malpractice to allow a patient to lie on her back; one or the other side or lying on the face should be resorted to to keep the uterus anterior. The primipara should be examined and measured three or four months before labor and should be again thoroughly inspected to see if the work is complete a month after delivery. To relieve pain he frequently uses Hyoscine 1-200 gr., Morphine 1-8 gr. Chloroform too long continued is dangerous.

Dr. Barnett said that repair work is usually a failure because of lack of deep stitches.

Dr. Archibald Smith said chloroform in convulsions adds to degeneration of liver and kidneys.

Dr. Duncan said his rule is to advise exercise and he tells the patient that she will have a large child unless she takes the the abdomen. The vaginal secretions are not helped by douches prior to labor unless one has infection. In sewing the perinæum, the stitches should approximate the parts without being too tight.

Dr. J. H. Neall read a paper on the "Elimination of Meat from the Diet."

Dr. Duncan said that we could probably not succeed in getting everyone to stop eating meat; one can assimilate the necessary amount of food from meat better than he can from vegetables alone; more especially is this true of the fats. Meat abstainers are more likely to have Burra Burra.

Dr. Niles said that we need to have such papers, because the writer gives only one side of the question. All the nations which have accomplished things have been great meat eaters and that while the use of meat has doubtless been excessive, yet the intemperance of total abstinence was as bad as excessive use.

Dr. LeConte said that since the epoch-making work of Mettchnikoff much had been learned. There are cases when meat should be eliminated; such as catarrhal entero-colitis, but for everyone to stop eating meat is not to be thought of. We doubtless ought to diminish the quantity we consume, but it remains a fact that the strong nations have used meat and this is the empirical evidence of the good of that food.

Dr. Neall said that he did not believe in cutting out meat in every case, but is putting in substitutes when he can. He, himself, has not eaten meat in twelve years but he *does* eat eggs. The cook should know how to compound food as well as the scientist does his work in a laboratory. He thinks that he was saved from pneumonia this winter by his low arterial pressure due to the lack of animal food.

